# Health systems and climate change mitigation through an applied health informatics and sustainability perspective

**DOI:** 10.1038/s44401-026-00157-1

**Published:** 2026-10-21

**Authors:** Mostafa Balboul, Manijeh Berenji, Asma Jahan Pathan, Muhammad Muinul Islam, Arriel Benis, Beenish Chaudry, Zerina Lokmic-Tomkins, Chethan Sarabu, Prerna Dua, Md Shafiur Raihan Shafi, Sarah Balboul, Titus Schleyer

**Affiliations:** 1 Hunterdon Medical Center, Flemington, NJ USA; 2 Rochester Regional Health, Rochester, NY USA; 3 Department of Medicine, Division of Occupational and Environmental Medicine, UC Irvine School of Medicine, Irvine, CA USA; 4 Department of Environmental and Occupational Health, Joe C. Wen School of Population and Public Health, University of California, Irvine, CA USA; 5 University of California Center for Climate, Health and Equity, Oakland, CA USA; 6 Health Informatics, University of North Carolina at Charlotte, Charlotte, NC USA; 7 Department of Biomedical Informatics, Biostatistics, and Medical Epidemiology, University of Missouri School of Medicine, Columbia, MI USA; 8 Truman School of Government and Public Affairs, University of Missouri, Columbia, MI USA; 9 Department of Biomedical Engineering, Duke University, Durham, NC USA; 10 Department of Biomedical Informatics, University at Buffalo, Buffalo, NY USA; 11 School of Computing and Informatics, University of Louisiana at Lafayette, Lafayette, LA USA; 12 School of Nursing and Midwifery and Health and Climate Initiative, Monash University, Clayton, VIC Australia; 13 Health Tech Hub, Cornell Tech, Cornell University, New York, NY USA; 14 Department of Pediatrics, Weill Cornell Medicine, Cornell University, New York, NY USA; 15 Department of Biomedical and Health Informatics, University of Missouri–Kansas City, Kansas City, MI USA; 16 Department of Internal Medicine, St. Luke’s University Health Network, Easton, PA USA; 17 Center for Biomedical Informatics, Regenstrief Institute, Inc., Indianapolis, IN USA; 18 Division of General Internal Medicine and Geriatrics, Department of Medicine, Indiana University School of Medicine, Indianapolis, IN USA

**Keywords:** Business and industry, Climate sciences, Developing world, Environmental sciences, Environmental social sciences, Health care, Mathematics and computing, Scientific community

## Abstract

Climate change poses a growing threat to U.S. healthcare systems, which are uniquely positioned to mitigate its impacts while addressing their own greenhouse gas emissions from energy use, supply chains, clinical operations, and waste. Clinical informaticists can advance mitigation by integrating environmental data into health system operations and decision-making. Tracking emissions across scopes enables data-informed strategies, while frameworks such as I-CLIMATE and One Digital Health can help coordinate disparate data streams and reduce healthcare’s environmental impact. Integrated dashboards can support renewable energy adoption, energy efficiency, and operational decision-making, while telehealth can reduce transportation-related emissions. Artificial intelligence may also reduce resource use when applied judiciously through narrowly scoped, task-specific models, with frameworks such as SAHAI guiding sustainable implementation. Financial approaches such as New York University’s Return on Sustainability Investment framework can quantify the financial value of decarbonization initiatives. Effective governance, including vertical alignment and horizontal integration, is essential to operationalizing these strategies. Emerging opportunities include climate-conscious hardware design and carbon accounting applications. Ultimately, informatics can provide the data, tools, and governance infrastructure needed to translate climate mitigation goals into measurable health system action and establish carbon neutrality as an organizational performance priority.

## Introduction

At the end of 2025, University of California (UC) Irvine Health’s newest hospital in Irvine opened its doors as the nation’s first all-electric acute-care hospital, marking a historic milestone in environmentally sustainable healthcare infrastructure and continuing the legacy of pioneers such as Gundersen Health System^1,2^. Powered entirely by renewable electricity — including rooftop solar arrays, a clean central utility plant, and fully electric heating, hot water, sterilization, and kitchen systems — the 144-bed, seven-story facility demonstrates that high-intensity, 24/7 patient care can be delivered without reliance on fossil fuels^3^.

The development of this “hospital of the future” reflects a broader paradigm shift in healthcare operations. As hospitals typically consume massive amounts of energy for heating, cooling, sterilization, and operations, the all-electric design at the UC Irvine Health hospital demonstrates that decarbonization at scale is both possible and practical. The hospital’s onsite solar system, procured through a power purchase agreement, generates approximately 10-15% of its energy demand and provides backup power, while offsite renewable electricity supplies the remainder of the estimated 13 megawatt-hours of annual energy needs. This example is a part of a broader movement in which leading institutions and health systems across the country are achieving the cost and societal benefits of environmental sustainability while improving operational and financial performance.

One may wonder what benefits would warrant such an approach to hospital design. The reality of the situation is that a climate-conscious approach leads to improved efficiency and sustainability of healthcare operations, reduced costs and improved financial returns, and healthier patients with decreased utilization and unnecessary hospitalizations^4,5^. In addition, by reducing reliance on fossil fuels, hospital systems become more energy-independent and mitigate the effects of energy supply and price volatility. On a larger scale, biodiversity loss and ecosystem collapse pose substantial threats to national security, geopolitical stability, food security, and many other facets of societal function both in and out of the context of healthcare; thus, mitigation of climate harm by healthcare facilities serves to keep communities and their ecosystems healthy, resilient, and stable^6^. Clinical informaticists are poised to help health systems reach these objectives, and in so doing help health systems stay profitable and efficient while concomitantly better serving their patients and communities. However, with the breadth of existing high-impact initiatives, from frameworks to direct interventions, combing through the volume of work and identifying new high-impact initiatives can be cumbersome.

To bridge this literature gap, we have scoped and compiled a list of areas where clinical informaticists have helped health systems realize the benefits of environmental sustainability, both those that are well-characterized and those with potential for further characterization, and corroborated with cases illustrating the success of these measures. The scope is indeed sizable, so we have bifurcated the compilation between the domains of Mitigation and Adaptation. This paper addresses the ways in which health systems should aim to mitigate emissions and waste, and in the process reduce harm to the immediate environment and the climate at large; the subsequent paper covers the means through which health systems can adapt to a changing climate and maintain resilience as they support their communities. To this effect, this paper presents insights from informatics that have demonstrable benefits for patients, staff, and health systems as a whole.

In this paper, we set out to acquaint our readers with the scope of informatics applications to environmental sustainability initiatives, with an emphasis on mitigation. With knowledge and experience from leading institutions and health systems across the country, we present both new and established methods for organizational leaders to achieve the cost and societal benefits of environmental sustainability by employing frameworks intended to drive operational success. These methods are corroborated with case studies illustrating real-world results achieved by organizations that have successfully implemented carbon-neutral operations. We also highlight areas for exploration, where the literature has room for characterization. The role of healthcare sustainability informatics and how it can be incorporated effectively into organizational structure is addressed, concluding with a look at future directions and global perspectives.

**Fig. 1:**
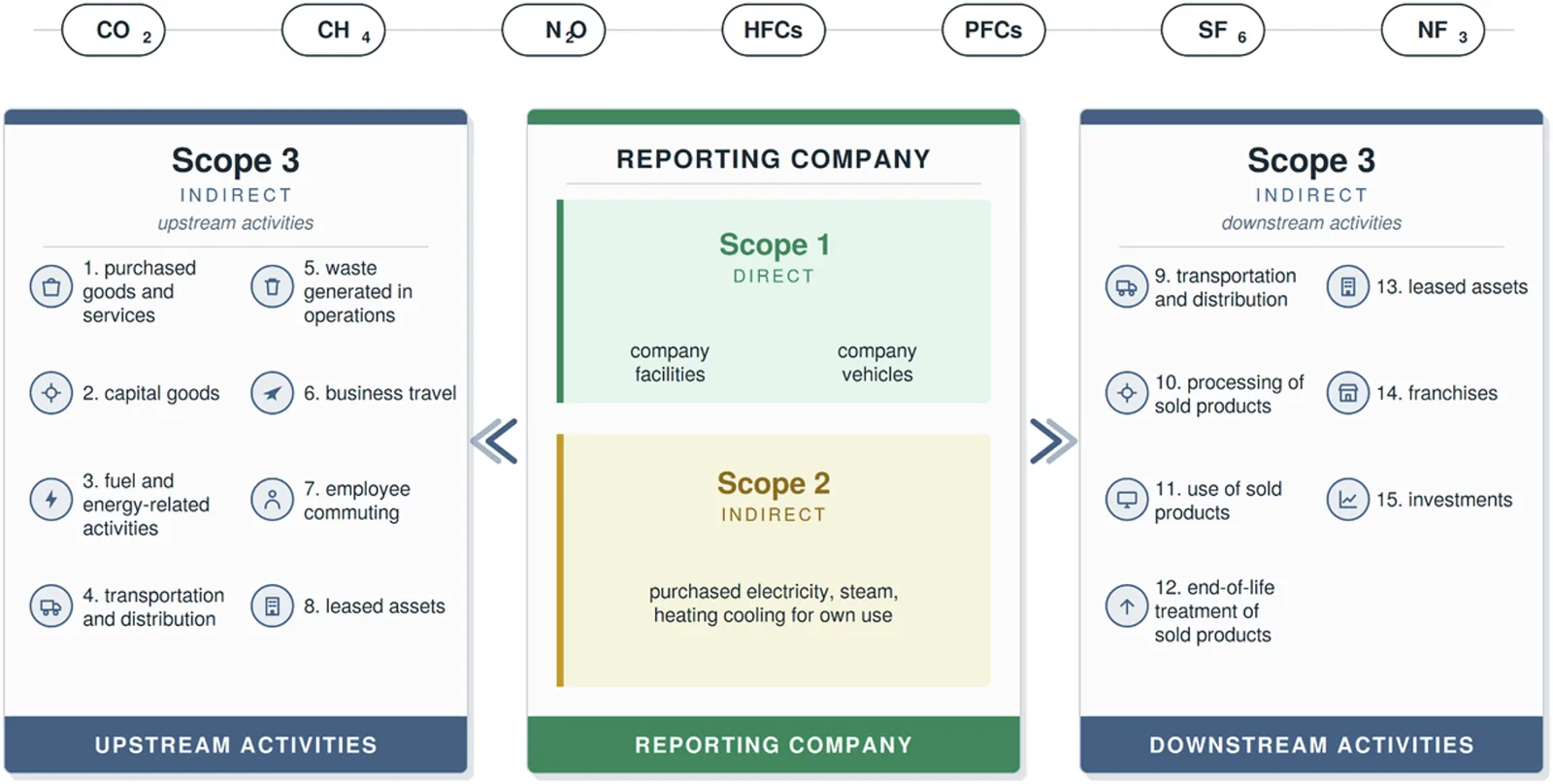
Schematic overview of greenhouse gas (GHG) emission Scopes 1–3 for health systems, organized by position along the value chain. Adapted from the World Resources Institute (WRI) and World Business Council for Sustainable Development (WBCSD) Corporate Value Chain (Scope 3) Standard.

Healthcare is a material contributor to GHG and a frontline responder to climate-related health sequelae. In the United States, economy-wide modeling estimated that the healthcare sector accounted for roughly 8.5% of national GHG in 2018^7^. In a global context, a widely cited assessment estimated that healthcare contributes about 4.4% of worldwide emissions^8^. More recent Lancet Countdown reporting suggests that the global health-sector footprint varied in recent years (e.g., ~5.2% in 2019 and ~4.2% in 2022), consistent with pandemic-era fluctuations and methodological differences across global accounting approaches^9–11^. The same global assessment estimates the United States healthcare footprint at roughly 546 Mt CO_2_e out of a global total near 2 Gt CO_2_e, which is approximately one quarter of global healthcare sector emissions^8^. These magnitudes motivate a mitigation agenda that is operational, measurable, and scalable across Scope 1, Scope 2, and Scope 3 emissions: Scope 1 covers direct emissions from sources owned or controlled by the organization (e.g., on-site combustion and fugitive releases), Scope 2 covers indirect emissions from purchased energy (e.g., electricity and heating/cooling), and Scope 3 covers other upstream and downstream value-chain emissions that are often dominated by purchased goods and services and therefore largely procurement-driven^12–14^. In the healthcare sector, approximately 83% of emissions are from Scope 3, with Scopes 1 and 2 comprising the other 17%^15^. To support consistent interpretation, we summarize these scopes and illustrative health-system emissions sources in a schematic overview organized by position along the value chain (Fig. 1)^16^. This perspective focuses on mitigation in delivery systems, with particular attention to the informatics, governance, and financial mechanisms that translate decarbonization commitments into routine clinical and operational decisions.

The three columns correspond to the segment labels beneath them. Upstream Activities (left): indirect Scope 3 emissions from supplier-side sources (categories 1–8, e.g., purchased goods and services, capital goods, business travel, employee commuting). Reporting Company (center): emissions associated with the reporting health system itself, comprising direct Scope 1 emissions from owned or controlled sources (e.g., facilities and vehicles) and indirect Scope 2 emissions from purchased electricity, steam, heating, and cooling for own use. Downstream Activities (right): indirect Scope 3 emissions occurring after the organization’s operations (categories 9–15, e.g., use and end-of-life treatment of sold products, investments). The seven Kyoto-basket gases covered by the GHG Protocol are shown at the top.

In the United States, policy signals and field initiatives have encouraged health systems to move from general sustainability commitments toward defined targets and reporting practices. Federal prioritization of climate and health activities varies across administrations. The White House and HHS Health Sector Climate Pledge, launched in 2022, outlines voluntary commitments that include a 50% emissions reduction by 2030 and net zero by 2050, with executive accountability, annual public reporting, and completion of a Scope 3 inventory by the end of 2024^17^. Recent changes in the visibility and accessibility of some federal climate and health resources indicate that the policy environment is dynamic and should be interpreted accordingly^18,19^. Complementary efforts such as the National Academy of Medicine Climate Collaborative emphasize measurement, transparency, and shared action across organizations^20^. Many mitigation measures remain justified on operational grounds regardless of political context because they can reduce energy and materials costs, limit exposure to energy price volatility and supply disruptions, and support enterprise risk management and quality governance. Internationally, the National Health Service England has published a net zero pathway and delivery plans, reinforcing that decarbonization is increasingly treated as a performance and governance priority rather than a peripheral environmental program^21^.

Drawing on our scoping synthesis of peer-reviewed literature, sector footprint assessments, and policy and standards documents cited in this section, we identify four recurring barriers to implementation. First, many organizations lack standardized, granular emissions data that can be attributed to facilities, service lines, and care pathways in a way that supports operational decisions. Measurement is often episodic or limited to high-level inventories, rather than integrated into routine management systems. Second, Scope 3 remains difficult to manage because supplier disclosure and product-level carbon data are inconsistent and not easily comparable. This opacity is consequential because the global healthcare footprint is dominated by supply chain emissions^8^. Third, the evidence base for prioritization is uneven. Leaders routinely face choices among infrastructure upgrades, clinical practice changes, procurement reforms, and digital investments, yet comparative evaluations that integrate emissions effects with financial returns and implementation feasibility remain limited. Fourth, virtual care reduces travel-related emissions, but system-level net effects depend on substitution versus addition of visits, utilization patterns, and the energy and material footprint of digital infrastructure. A recent scoping review synthesizing carbon footprint studies reports substantially lower emissions for virtual consultations than for in-person visits in many scenarios, while also highlighting heterogeneity in methods and limited evidence on net system effects as digital care scales^22^.

Sustainability informatics connects targets, implementation, and accountability by defining the data elements needed for Scope 1, 2, and 3 measurement, integrating those data streams into existing operational systems, and translating them into decision-oriented outputs that can be monitored over time. In practice, this includes automated capture and harmonization of facility energy and fuel data, procurement and supply chain data, and relevant clinical activity data, followed by attribution to facilities, service lines, or care pathways where feasible, and presentation through auditable dashboards that support routine management and external reporting. The i-CLIMATE framework provides a practical structure for these functions by specifying informatics roles that enable measurement, support environmentally preferable decision-making in clinical and administrative workflows, and strengthen governance through transparent metrics and feedback loops that inform policy and operational priorities^23^.

Health system sustainability is a community-shared mandate, rooted in the “Sustainability is everyone’s job” principle. Informatics, as embodied by the One Digital Health^24^ (ODH) framework, acts as the connective tissue and operational backbone, linking environmental and health system performance^25^ through integrated data across operations, supply chain, and patient care. ODH provides a unifying architecture where clinical, behavioral, environmental, educational, and logistical data converge to drive coordinated decision-making^26^. By positioning itself at the intersection of these paradigms, ODH functions as the operational engine that translates the broad ecological mandates of Planetary Health and the interspecies focus of One Health into a unified, data-driven architecture for health system sustainability. Embedding environmental metrics into interoperable digital ecosystems enables health systems to monitor GHG outputs, optimize resource flows, and build adaptive capacity with predictive tools^27^, following FAIR (Findable, Accessible, Interoperable, and Reusable) principles^28,29^. The FAIR principles treat data, metadata, and computational agents as entities able to locate, access, interoperate with, and reuse data with minimal human intervention. Sustainability in health systems rests on two pillars: reducing the ecological footprint through mitigation and building resilience through adaptation. Ultimately, informatics through ODH transcends its role as a technical utility to become a core governance engine; it facilitates the real-time synthesis of environmental intelligence and clinical workflows, allowing health systems to autonomously align ecological responsibility with system resilience through data-driven, adaptive decision-making.

European initiatives increasingly use ODH to structure data and information systems for climate‑sensitive health challenges at multiple levels, from urban ecosystems to hospital practice. In an urban freshwater project^30^, ODH guides FAIR‑aligned integration of environmental, human, and animal health data so that municipalities can detect emerging risks and coordinate ecosystem and public health interventions. In France, ODH‑inspired approaches inform projects that link climate hazards such as heat waves with chronic diseases like diabetes, using integrated clinical, environmental, and biosensor data and advanced analytics to support hospital decision‑making, protect vulnerable patients, and adapt care pathways during extreme events.

This Perspective highlights clinical informatics applications that support environmental sustainability in health systems, with emphasis on mitigation. Drawing on the authors’ professional experience across multiple U.S. health systems and on published evidence, we synthesize both established and emerging approaches that organizational leaders can use to operationalize decarbonization while attending to costs and broader societal benefits. The paper is organized around a practical framework intended to support implementation, measurement, and accountability. We corroborate key approaches with brief case examples that report real-world implementation and measurable outcomes. The intent is not to provide an exhaustive review. Instead, we highlight the informatics capabilities, governance arrangements, and evaluation considerations that recur across settings, and we identify areas where the evidence base remains limited, or reporting is not comparable. We conclude by outlining priorities for further characterization and describing implications for organizational strategy and global directions in healthcare sustainability informatics.

## Results

### Healthcare sustainability informatics

Healthcare sustainability informatics applies health data, information systems, and informatics methods to measure healthcare’s environmental impacts and support efforts to reduce them^23^. Existing frameworks establish the broad range of roles that informatics plays in this process. The i-CLIMATE framework, for example, identifies informatics-enabled actions related to circular and energy-efficient health information technology, environmentally informed clinical and administrative decision-making, workforce engagement, and environmental policy^23^. The Green-MIssion framework emphasizes how green medical informatics solutions support environmental monitoring, awareness, and interventions while considering technical, functional, process, and governance dimensions of implementation^23^. A common function of informatics across these frameworks is to move environmental information from measurement toward action. Drawing on the evidence reviewed here, we organize this process as an information-to-action cycle: environmental impacts are measured and used to inform decisions, those decisions guide changes in care delivery or operations, and resulting environmental performance is monitored over time (Table 1). System integration and equitable access shape implementation across this cycle. Our review shows that evidence is strongest for telehealth-based care substitution and anesthetic emissions management, while the direct environmental effects of broader informatics-enabled operational optimization remain less established.

**Table 1:** Healthcare sustainability informatics-based implementation strategies supporting climate mitigation in health systems

Information-to-Action Stage	Sustainability Informatics Objective	Representative Informatics Functions	Representative Evidence
Measure and Inform	Quantify and visualize healthcare’s environmental impacts to identify mitigation opportunities	Environmental metrics, EHR integration, automated data capture, dashboards, benchmarking, sustainability reporting	Sittig et al.^23^, Smith et al.^26^, Lam et al.^32^, Eckelman et al.^31^.
Intervene	Support lower-impact clinical decisions and care pathways	Telehealth care substitution, clinical decision support, workflow-integrated sustainability interventions	Muschol et al.^33^, Ramaswamy et al.^35^.
Cross-cutting Implementation	Enable sustainability informatics across healthcare organizations	System integration, interoperability, governance, workflow integration, stakeholder engagement, equitable access	Sijim-Eeken et al.^36^, Gallegos-Rejas et al.^37^, Mathew et al.^34^.
Evaluate and Adapt	Monitor environmental performance and continuously improve interventions	Longitudinal monitoring, dashboards, benchmarking, provider feedback, quality improvement	Kahn et al.^38^, Lam et al.^32^, Eckelman et al.^31^.

### Measure and inform

The first function of sustainability informatics is to make environmental impacts measurable and visible. Clinical, operational, and environmental data can be integrated to estimate emissions and resource use associated with healthcare services, encounters, and care pathways^23,26^. Existing frameworks further emphasize that sustainability metrics should be standardized, reliable, meaningful, integrated with health information systems, and aligned with the core mission of healthcare to support meaningful decision-making rather than simply reporting environmental performance^23^.

Lam et al., for example, integrated 24 pediatric anesthesia sustainability metrics into a native Electronic Health Record (EHR), enabling automated data capture, benchmarking, and provider- and department-level reporting without requiring separate manual data collection^23^. Aggregating these measures across providers, departments, and time provides a broader understanding of environmental performance and helps identify high-impact activities that may not be apparent from individual clinical encounters^23,26^. Dashboards, benchmarking, and reporting mechanisms make this information available to clinicians, administrators, and sustainability teams, enabling organizations to prioritize mitigation opportunities and monitor progress over time^26,31,32^. Sustainability informatics therefore establishes the information base required for environmental decision-making.

### Intervene

Sustainability informatics also supports changes in care delivery and resource use. The strongest evidence comes from care pathway substitution, particularly telehealth. When virtual consultations replace rather than supplement in-person visits, travel-related greenhouse gas emissions can be substantially reduced^33^. In a randomized controlled study of orthopedic and trauma follow-up care, video consultations avoided approximately 11.25 kg CO₂e per patient through avoided travel^33^. The magnitude of these benefits depends on travel distance, whether virtual care substitutes for face-to-face care, and the environmental footprint of the supporting digital infrastructure^33,34^.

Informatics can also influence how resources are used within existing clinical workflows. Ramaswamy et al. describe the implementation of a portable sustainability-oriented clinical decision support (CDS) toolkit that combines clinician education, hardware integration, software development, automated reporting, and real-time decision support to encourage lower fresh-gas-flow anesthesia practices^35^. Rather than replacing a clinical encounter, this approach uses continuously collected clinical data to influence decisions during care delivery while simultaneously supporting monitoring and quality improvement. This illustrates how sustainability informatics can actively shape clinical practice rather than merely measuring environmental performance^35^.

Informatics also has the potential to support more efficient healthcare operations through analytics, predictive modeling, scheduling optimization, inventory management, and resource planning. Although these applications may improve operational efficiency, relatively little empirical evidence currently demonstrates corresponding reductions in energy consumption, material use, waste generation, or greenhouse gas emissions. Consequently, operational optimization represents a promising but still emerging application of healthcare sustainability informatics.

### Cross-cutting implementation considerations

Healthcare sustainability informatics relies on information distributed across EHRs, scheduling systems, procurement and pharmacy records, operational databases, and analytics platforms^23,26^. Integrating these heterogeneous information sources enables environmental measurement, identification of mitigation opportunities, implementation of interventions, and ongoing monitoring of outcomes. The Green-MIssion framework similarly emphasizes that successful implementation requires attention to technical infrastructure, functional capabilities, organizational processes, governance, and stakeholder engagement^36^. System integration therefore provides the infrastructure through which sustainability informatics functions operate across healthcare organizations.

Equitable access represents a second cross-cutting implementation consideration, particularly when informatics changes how patients receive care. Telehealth studies consistently identify disparities related to socioeconomic circumstances, language, disability, digital literacy, internet connectivity, and technology access^34,37^. These factors influence who benefits from digitally enabled care and therefore shape the environmental and societal impacts of telehealth interventions. Consequently, although telehealth represents one of the strongest examples of informatics-enabled emission reduction, its implementation must also address barriers to equitable access^34,37^.

### Evaluate and adapt

The final function of the information-to-action cycle is determining whether mitigation efforts produce meaningful and sustained environmental improvements. Informatics supports this function through longitudinal measurement, dashboards, benchmarking, auditing, and feedback^23,26,36^. Sustainability metrics should be standardized, linked to healthcare performance, integrated with clinical information systems, and designed to support continuous organizational learning rather than one-time reporting^23^.

Evidence from anesthesia provides particularly strong examples of this evaluation process. In a two-year prospective performance-improvement investigation involving 53,294 patients and 79 anesthesiologists, Kahn et al. used automated data extraction and individualized monthly practitioner dashboards to monitor anesthetic greenhouse gas emissions and fresh-gas-flow practices^38^. Dashboard feedback was associated with reductions in median fresh-gas flow as well as overall anesthetic greenhouse gas emissions, demonstrating how continuous measurement and individualized feedback can support sustained improvements in clinical practice^38^.

Hence, the literature demonstrates several complementary roles for healthcare sustainability informatics throughout the mitigation process: measuring and making environmental impacts visible, integrating sustainability metrics into routine clinical information systems, informing clinical and operational decisions, supporting lower-impact care delivery, and monitoring environmental performance over time. These functions form an information-to-action cycle in which measurement informs intervention and subsequent evaluation provides evidence for continuous improvement. Current evidence is strongest for telehealth-based care substitution and anesthetic emissions management, whereas the direct environmental benefits of broader operational analytics and AI-enabled optimization remain an important area for future research.

### Energy and infrastructure

Hospitals and clinics operate continuously and require substantial energy for heating, cooling, ventilation, lighting, and medical equipment. These demands make the built environment an important source of healthcare-related GHG emissions, particularly in older or inefficient facilities where heat loss, excessive ventilation, outdated equipment, and inefficient lighting increase energy consumption^39^. Mitigation therefore involves two complementary strategies: reducing the carbon intensity of the energy supplied to healthcare facilities and reducing the amount of energy those facilities consume.

Transitioning to lower-carbon and renewable energy reduces emissions associated with healthcare facility operations. Although renewable energy technologies have environmental impacts across their life cycles, their operational GHG emissions are substantially lower than those of fossil-fuel-based energy sources. Informatics supports this transition through energy-management systems that track generation and consumption, compare renewable and non-renewable energy use, and monitor environmental and financial performance^40^. These systems provide health systems with information about whether renewable-energy investments are meeting expected energy, emissions, and financial targets.

Reducing energy demand provides a complementary mitigation pathway. Building-management systems, Internet of Things (IoT) sensors, and real-time energy monitoring provide information about energy use across Heating, Ventilation, and Air Conditioning (HVAC), lighting, and equipment and help identify inefficient operating conditions^41^. These systems also support automated control of building operations, such as adjusting heating, cooling, ventilation, and lighting in response to occupancy and environmental conditions. In this way, informatics links facility-level measurement with operational changes intended to reduce unnecessary energy consumption while maintaining the environmental conditions required for patient care.

Gundersen Health System (GHS; now Emplify Health) in Wisconsin illustrates how renewable-energy investments and energy-efficiency measures can operate together at health-system scale. GHS became the first U.S. health system to achieve energy independence in 2014 after implementing a portfolio of renewable-energy and efficiency initiatives across its facilities^1,2^. The health system reported a 40% reduction in energy use and approximately $1.2 million in annual savings, while energy monitoring and performance analytics supported tracking of facility consumption and progress toward its environmental goals^1,2^. The case illustrates the combined environmental and financial benefits associated with facility decarbonization, although capital requirements, technical capacity, and organizational commitment remain important implementation challenges.

### Supply chain and waste reduction

Sustainable transformation of healthcare systems increasingly depends on rethinking both the supply chain practices and the generation of surgical waste. In the context of this manuscript, “supply chain” refers to the upstream procurement and sourcing decisions that determine what products enter a health system in the first place, rather than solely how their waste products are managed. These decisions include vendor selection, product bundling and packaging, and the standardization of purchased goods across departments and service lines. Supply chain optimization therefore complements downstream waste reduction efforts by preventing unnecessary material from entering clinical workflows in the first place, which consequently reduces costs and associated emissions.

The operating room (OR) provides a particularly important setting for such interventions because of its resource-intensive practices and substantial contribution to hospital waste. Hospitals generate millions of tons of regulated and unregulated medical waste annually, with approximately one-third originating from ORs^42,43^. In 2020, the United States generated an estimated 5.9 million tons of medical waste, of which around 30% were from plastic materials^44^. These figures illustrate the scale of the opportunity for interventions targeting both the materials entering clinical environments and their management after use.

Several initiatives demonstrate the environmental and financial benefits of improving waste segregation and material recovery. Although much healthcare solid waste is potentially recyclable when uncontaminated and only a relatively small proportion is infectious, more than 50% may be classified as biohazardous waste. Most solid waste is recyclable if uncontaminated, and only a small part is infectious^45^. In 2021, the University of Turin in Italy implemented a waste separation program targeting non-contaminated and biohazardous waste. The initiative reduced the number and weight of biohazardous containers, increased recycling, lowered environmental impact, and saved approximately $4,135 in biohazard processing and incineration costs during the pilot study^45^. Similarly, the feasibility of a circular healthcare economy was demonstrated through the recovery and reuse of stainless-steel surgical instruments across a health system in the Netherlands. Through repair and recycling, the initiative generated savings of $46,097 while diverting more than a ton of metal waste from disposal, demonstrating how material circularity can contribute to both fiscal and environmental resilience^46^.

Other interventions demonstrate that sustainability opportunities extend further upstream to decisions about which products are purchased, how they are bundled, and from whom they are sourced. At a pediatric hospital in the U.S., the redesign of surgical supply packs to remove infrequently used disposable items eliminated nearly 2 tons of OR waste and generated more than $27,000 in annual savings without compromising patient care, quality standards, or workflow efficiency^47^. The same initiative identified an irrigation device used by otolaryngologists that required four separately purchased components from different suppliers. Consolidating these components under a single supplier generated an additional $70,000 in annual savings, independent of changes to intraoperative waste handling. Importantly, the initiative leveraged the EHR to track opened and unused sterile supplies from preformed surgical packs, illustrating how existing health information systems can help identify opportunities for both waste reduction and supply chain optimization.

Product substitution provides another mechanism for reducing the environmental burden associated with surgical supplies. Disposable “blue wrap” used to sterilize surgical instruments contributes an estimated 115 million kilograms of plastic waste annually in the U.S. alone. A life-cycle assessment comparing disposable wrap with multi-use rigid sterilization containers (RSCs) found substantial environmental advantages for reusable containers, with an environmental break-even point reached after approximately 60 use cycles. In one U.S. hospital, replacing disposable polypropylene sterilization wrap with reprocessed hard metal cases generated approximately $51,000 in annual savings^44^. Such findings demonstrate how procurement decisions that replace single-use products with durable alternatives can simultaneously reduce material waste and operating costs^48^.

The aforementioned initiatives demonstrate that healthcare sustainability can be advanced through multiple, interconnected strategies: improved waste segregation, material recovery and reuse, product substitution, supply-pack redesign, vendor consolidation, and more sustainable procurement. However, identifying and scaling these opportunities across complex health systems requires the ability to connect information about what is purchased, what is used, what remains unused, what is discarded, and the environmental and financial consequences of these patterns. Informatics provides the infrastructure for integrating and analyzing these data across procurement, clinical, inventory, and waste-management systems. Resource-management platforms and emerging analytical technologies, including AI, may further support demand forecasting, optimized resource utilization, and identification of inefficient purchasing and consumption patterns^49,50^. Integrating procedural and clinical data with procurement, inventory, maintenance, and waste data could therefore enable health systems to move from isolated sustainability initiatives toward systematic, data-driven supply-chain optimization, material circularity, and waste reduction.

### Financing and business case

The business case for healthcare sustainability, guided by informatics, centers on presenting environmental initiatives as strategic investments that yield measurable long-term financial returns alongside broader organizational and societal benefits. Informatics plays a crucial role in providing the data and analytics necessary to measure, track, and demonstrate the financial and health benefits of environmentally sustainable practices.

The“Information technology-enabled Clinical cLimate InforMAtics acTions for the Environment” (i-CLIMATE) provides the framework for how informatics can help reduce healthcare’s environmental pollution and climate-related impacts, utilizing five actionable components^23^ These include: (1) create a circular economy for health IT, (2) reduce energy consumption through smarter use of health IT, (3) support more environmentally friendly decision-making by clinicians and health administrators, (4) mobilize healthcare workforce environmental stewardship through informatics, and (5) inform policies and regulations for change. The authors define Clinical Climate Informatics as a field that applies data, information, and knowledge management principles to operationalize components of the i-CLIMATE framework.

Building on i-CLIMATE, a cost-benefit analysis (CBA) assesses both the direct and indirect impacts of a project, comparing costs to benefits^51^. CBA consists of the following components: (1) implementation costs to fund initial investment in new technology or infrastructure; (2) operational costs related to new processes, maintenance, or staff training; and (3) IT-related costs (including software, analytics platforms, and data integration) to assess and monitor project impact. In projects designed to mitigate the effects of climate change, CBA can optimize work to improve resource allocation, enhance project effectiveness, and achieve sustainable and resilient results^52^. Specialized informatics tools (such as sustainability management platforms, carbon accounting and abatement planning suites, Material Flow Cost Accounting systems, and life‑cycle costing software) can help track and quantify the costs associated with environmental sustainability initiatives^53^. An informatics-driven CBA can highlight these returns by quantifying the factors responsible for the benefit. A recent review concluded that CBA is a robust decision tool for assessing sustainable upgrades in existing buildings, with energy savings most commonly quantified^54^. The synthesis mapped methods, cost modeling approaches, benefits considered, recent trends, and research gaps, underscoring the value of CBA despite data and methodological constraints. Applied to healthcare facilities, the same framework can rank hospital retrofits (HVAC, lighting, insulation, water) by monetizing utility savings, maintenance impacts, and emissions reductions alongside reliability and resilience benefits.

Expanding on CBA, the NYU Stern Center for Sustainable Business’ Return on Sustainability Investment (ROSI™) Healthcare Delivery Systems Decarbonization Framework enables healthcare systems to cut emissions by monetizing decarbonization investments, organizing eight strategies (energy management, building design and maintenance, sustainable sourcing, food service optimization, low‑emissions clinical care, waste reduction, responsible investments, and reduced transportation) under leadership, financing, and education pillars to unlock financial value and resilience^55^. The NYU team has applied this framework for Advocate Health, enabling them to rigorously capture and monetize the clinical, environmental, and financial value of switching from single-use to reprocessed medical devices^55^. ROSI was used to identify tangible and intangible benefits across operational efficiencies, supplier relations, and stakeholder engagement, with four benefits in operational efficiency and risk management monetized via data collection and financial modeling. The analysis demonstrates a net positive return, with a 10-year Net Present Value of $20.3 M (present value of total benefits $20.8 M less program costs $0.5 M) and an average annual net benefit of $3.5 M, approximately 3% of Advocate Health’s 2023 operating income, while delivering measurable emissions reductions of 1.5 kg CO_2_e per reprocessed device and an average annual reduction of 857 metric tons CO_2_e over the decade. As an HHS Pledge signatory operating across a large, multisite enterprise, Advocate Health demonstrates how incorporating informatics-embedded decarbonization into strategic decision-making by quantifying risk reduction, operational efficiency, and stakeholder value accelerates the adoption of high-impact reprocessing programs while maintaining transparency on data quality and context.

The following case studies illustrate how health systems have put some of the concepts described above into practice.

### Health system case studies

#### Case Study: Indiana University Health - Building a systemwide culture of sustainability

Indiana University Health (IU Health), Indiana’s largest healthcare system and its only academic health center, has made sustainability a strategic priority, grounded in its vision to make Indiana one of the nation’s healthiest states. Recognizing that environmental stewardship directly affects community health, IU Health’s leadership has embedded sustainability within its 2025–2029 system strategic plan as one of five core commitments alongside clinical excellence, affordability, whole-person care, and academic mission.

IU Health’s sustainability journey began in 2020 with decentralized efforts, such as LEED-certified buildings and energy conservation projects, but accelerated after strong board and executive support reframed sustainability as a systemwide responsibility. Under CEO Dennis Murphy and Senior Vice President for Philanthropy and Sustainability Crystal Miller, IU Health established a formal Office of Sustainability in 2024. The structure now includes an Executive Director of Sustainability, an energy director, a project manager, and system-wide Sustainability Councils and Task Forces spanning energy, food, waste, procurement, transportation, and green building.

These coordinated efforts have led to measurable outcomes. In 2024, IU Health Methodist Hospital received Practice Greenhealth’s Partner Recognition Award, and by 2025 all 16 hospitals achieved Partner for Change awards, making IU Health one of only 15 systems nationwide to earn the System for Change Award. The organization also became one of the first ten health systems to receive The Joint Commission’s Sustainable Healthcare Certification. System initiatives include replacing Styrofoam in cafeterias, implementing healthy food guidelines, managing energy use in-house, and expanding single-use device reprocessing, saving over $2 million in costs while increasing staff engagement by 144% in sustainability challenges.

IU Health’s sustainability governance is deeply integrated. The Sustainability Executive Committee and Sustainability Council engage leaders from operations, nursing, clinical services, and community health, ensuring alignment with Practice Greenhealth benchmarks and U.S. Department of Energy’s Better Climate Challenge. Progress is tracked through a Power BI GHG dashboard covering energy use, anesthetic gases, fleet emissions, and inhaler use, creating transparency and accountability at both hospital and system levels.

Key enablers include visible senior leadership commitment, integration into performance improvement frameworks, and transparent communication through IU Health’s internal sustainability portal, SharePoint site, and regular staff updates. Challenges persist, particularly in aligning standards across a complex, multi-hospital system, but IU Health’s experience shows that embedding sustainability into culture, data, and governance can yield tangible results for environmental performance, fiscal stewardship, and community health.

#### Case study: Emplify Health (formerly Gundersen Health System)—Pioneering energy independence and sustainable healthcare

Emplify Health, formerly Gundersen Health System, has long been recognized as one of the first health systems in the United States to achieve energy independence, a milestone reached in 2014 through its comprehensive commitment to sustainability. Serving nearly 277,000 patients annually across Wisconsin, Minnesota, and Iowa, Emplify’s environmental initiatives are grounded in the belief that improving environmental performance directly advances the health of the communities it serves.

The organization’s sustainability program is led by Envision, a wholly owned subsidiary established in 2009 to manage and expand Emplify’ s environmental stewardship projects. Guided by an independent board of directors, Envision develops and oversees programs in energy efficiency, renewable energy generation, waste reduction, and sustainable construction. Through a combination of energy reduction, utility-scale renewables, and on-site generation, Emplify avoids approximately $5–6 million annually in energy costs. The system has invested in biomass, methane capture, geothermal, solar, and wind energy projects, significantly lowering its carbon footprint while providing a model for community-based clean energy partnerships.

Sustainability principles are embedded into new construction, remodeling projects, operations, and supply chain management. Educational programs engage staff, patients, and community members in sustainable practices, reinforcing a culture of environmental and fiscal responsibility. Leadership commitment, cross-departmental collaboration, and reinvestment of savings into new sustainability projects have been key to the program’s enduring success.

Despite the challenges of scaling second-generation initiatives amid tighter healthcare margins and regulatory complexity, Emplify continues to expand its sustainability agenda. Its journey demonstrates how a health system can translate clinical and community values into measurable environmental and financial benefits, reducing operational costs while improving public health and resilience.

#### Case Study: UC San Diego Health - Embedding sustainability into clinical and operational practice

UC San Diego Health has integrated sustainability as a strategic priority, grounded in the recognition that environmental stewardship is essential to both health equity and clinical quality. Serving more than half a million patients annually across facilities that include two safety net hospitals, UC San Diego Health views sustainability as central to protecting the health of vulnerable populations while advancing long-term system resilience.

Established in 2019, UCSDH’s sustainability program at UC San Diego Health builds on the University of California system’s broader commitment to environmental responsibility, formalized through the 2004 UC Sustainable Practices Policy. The program targets several domains: energy-efficient and LEED-certified infrastructure powered by 100% clean electricity; waste reduction through recycling and green operating room initiatives; sustainable food systems emphasizing plant-based, local, and organic options; and a system-wide goal of fossil-free operations by 2045. Additional initiatives focus on water conservation, toxin reduction, and climate adaptation in high-risk communities.

UC San Diego Health reports a range of benefits from these efforts. Energy use intensity has decreased ~8% between 2023 and 2024; over four years, the device collection program grew at a compound annual growth rate (CAGR) of 8%, generating savings with a CAGR of 15%, totaling $3.7 M, and a soft plastics recycling program has been established, diverting almost 2 tons of waste from the landfill. The new McGrath Outpatient Pavilion at Hillcrest Medical Center is designed to reduce carbon intensity by more than 90% and achieve LEED Silver certification. The organization also developed the first climate-resilient Hazard Vulnerability Assessment tool in the United States, accompanied by clinician toolkits that connect environmental exposures to patient care.

Sustainability governance is supported at multiple levels. Executive leadership — particularly Chief Executive Officer Patty Maysent and Medical Director of Sustainability Dr. Shira Abeles — has provided clear vision and accountability, while the sustainability team fosters cross-departmental collaboration. Of 14,000 employees, 3244 engage in“green clinic” certifications, team-based improvement projects, and sustainability huddles, reinforcing a culture of shared ownership.

Key success factors include visible leadership commitment, integration with quality improvement frameworks, transparent metrics, and alignment with broader institutional goals. Persistent challenges include financial constraints and the behavioral change required to embed sustainability in complex clinical systems. UC San Diego Health’s experience illustrates that sustained leadership attention and system-wide engagement can translate sustainability principles into tangible improvements in operational efficiency, environmental performance, and community health.

#### Case study: Rochester Regional Health - Cost-effectiveness and emissions reduction through Telehealth

Rochester Regional Health has prioritized minimizing healthcare’s carbon footprint, and that extends to its research. With over 17 million kilowatt-hours in annual energy efficiency savings and over 15 million pounds in annual greenhouse gas (GHG) emission reduction, Rochester Regional saves over $2.4 million annually through renewables, energy efficiency, and waste reduction initiatives, illustrating the success that results when the symbiosis between sustainability goals and financial goals is recognized.

7This commitment to environmental sustainability extends to its research. In the first year of the COVID pandemic, telehealth provided the majority of outpatient clinical care throughout the USA, and upstate New York was no exception. Rochester Regional Health’s Vice President of Health Informatics Matt Phillips presented on the emissions and cost reductions that resulted from this paradigm shift in ambulatory care, working alongside Dr. Kaiwen Zhu to analyze and quantify the benefits^56^.

Using a randomized subset of 2000 telehealth visits (out of the 512,683 total visits conducted between March 2020 and March 2021), they analyzed the emissions and cost reductions resulting from switching these visits from in-person. They used R programming with the Google API to calculate the travel distance and time for each visit, and used cost estimates from the US Department of Energy and New York State. Estimated cost was based on the IRS standard mileage rate and New York State’s minimum wage in 2021, and carbon impact was based on EPA estimates for CO_2_ emissions per gallon of gasoline. After removing outlier visits over 3 standard deviations from the mean, results illustrated a median of 13.39 miles saved per clinical visit, a median travel time of 0.43 hours, and an estimated gasoline savings of 0.55 gallons per visit. The median travel distance differed dramatically between departments, with the three highest median round-trip distances being in the Pain Medicine, Infectious Disease, and Orthopedic Surgery clinics, at 28.10 miles, 27.48 miles, and 25.94 miles, respectively. The cost savings are approximated at $13.95 per visit, and the carbon savings at 4.92 kilograms per visit. Extrapolating these figures to the entire annual patient cohort, the estimated annual savings from travel reduction comprise 8,957,607 miles, 239,126 hours, and $8,603,164; carbon savings total 3,289,515 kilograms, equivalent to the CO_2_ removal capacity of hundreds of acres of forested terrain.

Through analytics, the Health Informatics department has quantified the benefits of telemedicine through cost-effectiveness and emission reduction, and set the stage for wider adoption of telehealth solutions. Further exploration of telemedicine applications is being pursued, including decreasing infection spread and improving access for mobility-challenged patients. There may be a role for leveraging CDS in predicting when to recommend telehealth follow-ups for specialties with notably higher median travel distance. While there are limitations to consider for future studies, such as generalizing to other parts of the country, these results paint an auspicious picture for the role of telehealth in making ambulatory care more sustainable, and illustrate informatics’ role in making a business case for such initiatives through tangible demonstration of ROI.

## Discussion

Advancing healthcare sustainability requires governance models that extend beyond vertical leadership roles to enable coordinated, system-wide action. Many health systems have appropriately begun with top-down approaches, establishing executive accountability through Chief Sustainability Officers and formal governance committees. Over time, this structure has expanded to include clinically embedded roles, such as Medical Directors of Sustainability within domains like anesthesia and surgery, where emissions-intensive practices are concentrated. While these roles are essential for legitimacy and local ownership, experience increasingly demonstrates that durable emissions reduction cannot be achieved through siloed leadership alone. Effective mitigation requires both vertical alignment, from board-level strategy to frontline implementation, and horizontal integration across clinical, operational, supply chain, and digital domains^57^.

Informatics functions as a critical enabler of this integrated governance model. As a discipline centered on measurement, attribution, and decision support, informatics translates sustainability commitments into operational signals that can be embedded within routine workflows. These capabilities include granular emissions accounting, environmentally-informed clinical decision support (e.g., anesthetic gas selection or inhaler prescribing), and transparent dashboards that align sustainability metrics with quality, safety, and financial performance^26^. At the same time, digital systems themselves contribute to healthcare’s environmental footprint, necessitating explicit governance over health IT lifecycles and AI deployment. Frameworks such as i-CLIMATE and Sustainably Advancing Health AI (SAHAI) highlight that sustainability governance must therefore encompass not only what informatics enables, but also how digital infrastructure is designed, procured, and operated^58^. Positioning informatics as an instrument of decarbonization and an object of stewardship allows health systems to move from symbolic leadership structures toward accountable, data-driven mitigation embedded in everyday decision-making.

To maximize global impact and address significant differences in scale and resource availability, health systems should adopt a tiered sustainability implementation approach based on informatics capabilities to model, optimize, and implement sustainability processes and policies tailored to their components. This approach would ensure that every organization, from a single rural clinic to a multinational network, has a clear starting point and continuous mitigation to support system sustainability and address climate change^59^.

Large, integrated delivery networks (macro-level), such as the NHS or major US health systems, are uniquely positioned to drive market transformation. Their focus encompasses governance, supply chain standardization, and business innovation. These organizations use their extensive procurement capabilities and centralized data systems to enforce rigorous sustainability standards for external vendors, making compliance a contractual obligation^60^. The role of informatics at this level involves implementing interoperable data standards (e.g., the Fast Healthcare Interoperability Resources [also known as FHIR] extension for environmental data) to benchmark and aggregate emissions across numerous sites, translating system-wide metrics into actionable executive dashboards^27,61^. These reports would aim to transform health system infrastructures and resources, such as renewable energy power purchase agreements^45^.

Medium-sized healthcare centers (meso-level) concentrate on operational efficiency and scalable pilot initiatives. Their efforts are focused on internal enhancements to implement facility-specific resource management and process improvements. This encompasses optimizing operating room processes to reduce waste^45,47^ and implementing targeted circular economy pilots, such as reusable surgical trays^46^. Through informatics and AI, data flows directly into the EHR, allowing resources to be adapted to needs and making health systems sustainable by reducing unnecessary consumption^62^. Nevertheless, AI must be used responsibly to reduce the environmental impact of power consumption associated with these technologies^58^.

Small or community-based hospitals and clinics (micro-level) are healthcare environments emphasizing cost-effective innovation and community involvement^63^. Their primary focus is on generating immediate local impact, including establishing low-carbon local supply chains and using telehealth to reduce patient travel emissions. This approach mirrors innovative strategies observed in solar clinics within resource-constrained settings^64^. The aim of informatics in this context is to develop fundamental data collection mechanisms to monitor compliance and report key metrics, thereby enabling even the most modest facilities to contribute to the larger, harmonized data repository aligned with the One Digital Health vision. This tiered approach guarantees that every health system, no matter its size or location, has a clear entry point into climate mitigation, all unified by the shared need for reliable, comparable, and actionable environmental data provided by informatics.

As adoption of software employing AI scales rapidly, the importance of judicious selection of AI solutions becomes clear. While the landscape of health AI is likely to continue evolving beyond the scope of this paper, there exists a framework that is likely to provide organizational benefit in AI usage optimization. The Sustainably Advancing Health AI Framework (SAHAI) provides guidelines for measuring and mitigating the emissions, energy, and costs of AI implementations, while maintaining clinical performance and equity goals^60,65^. The SAHAI framework breaks down levers to reduce the environmental footprint of AI across multiple domains, starting with data centers, model reduction, application orchestration and overall policies of use. Within the data center section, the authors emphasized that data center efficiency, cleaner energy sources, and advanced cooling technologies can significantly reduce the environmental footprint of health AI, making vendor and infrastructure choices critical for sustainable implementation. There are now emerging transparency tools such as EcoLogits and AI Energy Score that aim to quantify and compare the energy and environmental impacts of AI models to support more sustainable decision-making. EcoLogits is a framework and open-source tool for estimating the energy use and environmental impacts of generative AI inference^66^. AI Energy Score, hosted through Hugging Face, is presented as a benchmarking and labeling initiative that evaluates and ranks AI models based on standardized measurements of energy consumption under controlled hardware conditions, with documentation and related technical reports outlining its methodology^67^.

Within the EHR, there is nascent research in addressing pharmaceuticals and food’s environmental impact through clinical decision support. The pharmaceutical industry has a sizable effect on the environment in its supply chain, from the resources used by the manufacturing process to the chemical byproducts requiring disposal^68^. On the part of health systems, the disposal of expired and partially used medications warrants scrutiny^69^. Food contributes to approximately 9% of healthcare’s carbon footprint in the US, including GHGs from ruminant meats, and food waste itself^70^. Plant-based diets and local supply chains have been shown to reduce emissions substantially, and hospital food systems are no exception. A new health care prevention agenda: Sustainable food procurement and agricultural policy^71–73^. For both nutrition and medications, clinical decision support systems have the potential to bolster efforts to reduce waste and emissions through dashboards tracking pharmaceutical inventory for data-driven strategies with the former, and integration of sustainability data with nutritional requirements for environmentally sustainable meal planning with the latter^74–76^.

In addition to software, climate-conscious hardware is also a promising horizon both within and beyond the context of healthcare. At many institutions, research is being conducted in computer architecture and systems design to improve the performance and efficiency of hardware solutions, and in the process, make them more carbon-neutral and less energy-intensive^77^. As more effort goes into creating energy-efficient hardware to power intensive computational tasks (such as those involved in health information technology), staying informed about environmentally-sustainable computing innovations and advocating for the use of clean energy in powering these innovations will enable healthcare systems to make key purchasing decisions that will achieve carbon neutrality goals and lower electricity costs^78,79^.

An often-overlooked but undeniably crucial facet of health information technology is cybersecurity; as cyber threats become more intricate and ubiquitous, security software becomes more energy-intensive. Green cybersecurity is a blossoming field that explores how to reduce the environmental impact of cybersecurity operations. A growing body of research is discovering how AI (including machine learning and large language models) can bolster cybersecurity practices by improving resource management, predicting threats more efficiently, and optimizing energy usage^80^.

The last of the future directions is one that comprises most of the GHG emission burden; Scope 3 represents 80% of GHG emissions from healthcare settings, but the characterization of informatics solutions for optimizing the supply chain (and Scope 3 emissions more broadly) is sparse. As with many other future directions, there is nascent recognition of the potential for AI to solve some of the inefficiencies and challenges of supply chain^81^; optimizing the supply chain will inherently reduce emissions, whether or not sustainability is the central objective. Aside from AI, health economists are poised to help track different facets of the supply chain and provide insight on actionable steps to reduce them, through their expertise in quantifying efficiency and value of healthcare provision; however, they rarely get the opportunity to address this particular subject. As health system leaders, we can help advocate for health economists to do this important work by creating positions and giving them the tools they need (such as the aforementioned carbon accounting software applications) Given the preponderance of promising horizons for informatics in mitigating climate change, a summary has been provided in Table 2.

**Table 2:** Horizons for clinical informatics supporting climate mitigation

Future direction	Subject matter	Barriers	Current state
Carbon accounting software applications	Carbon footprint evaluation and tracking	Adoption	Various solutions exist, others in development
Clinical decision support for nutritional selection	Food, Nutrition waste	Stigma around plant-based diets, lack of consistent policy frameworks	Interventions exist, not yet employing clinical informatics significantly
Clinical decision support for pharmaceutical selection	Pharmacology, Pharmaceutical waste	Understanding longitudinal effects, working on improving supply chain	Used for anesthetics, growing usage for inhalers, other use cases being characterized
Climate-friendly hardware	Computer science, Electrical and computer engineering	Predominance of traditional computer architecture	Subject to substantial research internationally as efficient computation is ubiquitously desired
Green Cybersecurity	Cybersecurity, Artificial intelligence	Counterbalancing energy expenditure of AI with improved efficiency of cybersecurity algorithms	Demonstrated potential through research, studies still underway
Plastic alternatives	Materials Science, Biology	Finding adequate candidates	Various solutions exist, others in development
Remote patient monitoring	Biochemistry, Biophysics	Solution procurement and integration with health systems	Various solutions exist, others in development
Supply Chain and Procurement	Health Economics	Lack of awareness, health economist positions, or tools to analyze relevant data	Some tools already exist, and AI’s role in supply chain is slowly being characterized

**Fig. 2:**
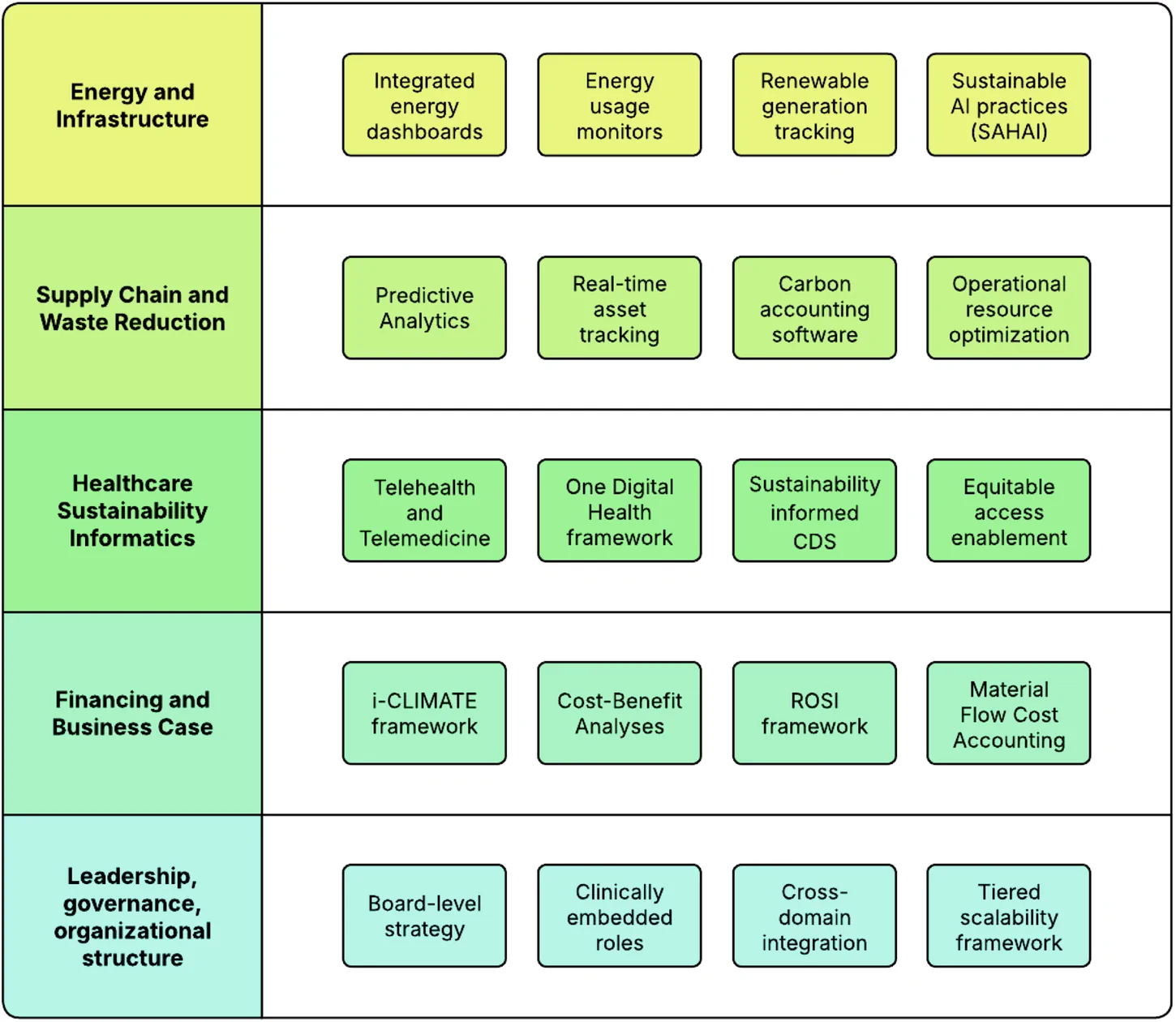
Informatics for mitigation: key intervention domains and strategies.

The role for healthcare sustainability informatics in achieving carbon-neutrality of operations is a powerful one, and multifaceted in scope as demonstrated by Fig. 2. The mitigation sector alone has many facets and areas for informatics to meaningfully address, notwithstanding the role of clinical informatics in addressing adaptation strategies. The benefits of mitigating harms caused by wasteful/inefficient operations and pernicious energy sources are similarly broad, from individual patients to their communities, not to mention the healthcare systems that serve them. In tracking and visualizing key metrics and operations, optimizing the supply chain and reducing waste, demonstrating the returns on investment with robust data collection, and taking leadership in addressing environmental sustainability, there are myriad opportunities within the informatics sector to mitigate climate change. With benefits ranging from increased efficiency to better patient outcomes, decreased waste, and improved finances, the reasons to run healthcare operations sustainably should be the rule, not the exception. The field of clinical informatics can advance these goals and simultaneously illustrate their benefits for organizations that adopt them. As previously mentioned, it is impossible to fathom every future direction for climate health solutions in healthcare, but by applying the frameworks as listed above, health systems should be able to stay at the vanguard of environmental sustainability initiatives and use them to achieve their collective targets. Sustained commitment to informatics-enabled mitigation will determine how effectively health systems can meet the accelerating climate challenges ahead; with that in mind, we wish you the best in operationalizing these insights and bringing success to your healthcare system.

## Methods

This Perspective presents a narrative review of the current state of climate change mitigation within U.S. health systems, focusing on the role of health informatics in supporting measurement, implementation, governance, and evaluation. This review examined relevant peer-reviewed literature, health system reports, policy and organizational guidance, and publicly available materials to identify established and emerging approaches to reducing the environmental impact of healthcare delivery. The review focused primarily on U.S. health systems and considered key domains including greenhouse gas emissions measurement, energy and resource management, telehealth, artificial intelligence, financial valuation, governance, and informatics-enabled sustainability strategies. Case studies were incorporated to illustrate how these approaches have been operationalized within health systems of varying sizes and organizational structures. The case studies are illustrative examples rather than a representative sample or a comparative evaluation of health system performance. Given the Perspective format, this work was designed as a narrative synthesis of the current landscape and practical applications and was not conducted as a systematic review or meta-analysis.

## Data Availability

No datasets were generated or analyzed during the current study.

## Code availability

No computational analyses or custom code were developed or used for this Perspective; therefore, code availability requirements are not applicable.
